# Genetics, Immunity and Nutrition Boost the Switching from NASH to HCC

**DOI:** 10.3390/biomedicines9111524

**Published:** 2021-10-23

**Authors:** Paola Dongiovanni, Marica Meroni, Miriam Longo, Silvia Fargion, Anna Ludovica Fracanzani

**Affiliations:** 1 General Medicine and Metabolic Diseases, Fondazione IRCCS Ca’ Granda Ospedale Maggiore Policlinico, Pad. Granelli, 20122 Milan, Italy; maricameroni11@gmail.com (M.M.); longo.miriam92@gmail.com (M.L.); silvia.fargion@unimi.it (S.F.); anna.fracanzani@unimi.it (A.L.F.); 2 Department of Clinical Sciences and Community Health, Università degli Studi di Milano, 20122 Milan, Italy; 3 Department of Pathophysiology and Transplantation, Università degli Studi di Milano, 20122 Milan, Italy

**Keywords:** NAFLD, NASH, heritability, HCC, nutrition

## Abstract

Nonalcoholic fatty liver disease (NAFLD) is the leading contributor to the global burden of chronic liver diseases. The phenotypic umbrella of NAFLD spans from simple and reversible steatosis to nonalcoholic steatohepatitis (NASH), which may worsen into cirrhosis and hepatocellular carcinoma (HCC). Notwithstanding, HCC may develop also in the absence of advanced fibrosis, causing a delayed time in diagnosis as a consequence of the lack of HCC screening in these patients. The precise event cascade that may precipitate NASH into HCC is intricate and it entails diverse triggers, encompassing exaggerated immune response, endoplasmic reticulum (ER) and oxidative stress, organelle derangement and DNA aberrancies. All these events may be accelerated by both genetic and environmental factors. On one side, common and rare inherited variations that affect hepatic lipid remodeling, immune microenvironment and cell survival may boost the switching from steatohepatitis to liver cancer, on the other, diet-induced dysbiosis as well as nutritional and behavioral habits may furtherly precipitate tumor onset. Therefore, dietary and lifestyle interventions aimed to restore patients’ health contribute to counteract NASH progression towards HCC. Even more, the combination of therapeutic strategies with dietary advice may maximize benefits, with the pursuit to improve liver function and prolong survival.

## 1. Introduction

Nonalcoholic fatty liver disease (NAFLD) is the leading contributor to the global burden of chronic liver diseases [1]. Its prevalence is approximately 25% ranging from 13% in Africa and 42% in southeast Asia and the hallmark of the disease is excessive fat deposition in hepatocytes [2]. NAFLD comprises a spectrum of histological conditions ranging from simple steatosis which is considered a benign as well as a reversible condition to nonalcoholic steatohepatitis (NASH) in which triglyceride accumulation in the hepatic parenchyma is associated with inflammation and ballooning [3]. NASH may progress to fibrosis, cirrhosis and hepatocellular carcinoma (HCC) and it represents the second most common indication for liver transplantation in the United States [4]. Indeed, recent advances in viral hepatitis therapies have been paralleled by the epidemic of obesity and type 2 diabetes (T2D), which to date mainly boost NASH progression up to HCC. Therefore, the growing burden of NAFLD is allied with the increasing incidence of HCC which represents the 75–85% of liver cancer and the sixth- most common tumor worldwide [4].

The annual incidence of NAFLD-related HCC in USA and Europe ranges from 0.7% to 2.6% in patients with NASH-related cirrhosis whereas it is lower (0.1 to 1.3 per 1000 patient-years) in non-cirrhotic NAFLD and the proportion of HCC attributable to NAFLD is higher in Germany, UK, India and Middle East [2]. NASH-HCC usually occurs in older patients, it is diagnosed at later stages and is associated with poorer survival compared to viral hepatitis-related HCC [2]. Moreover, it may develop also in the absence of cirrhosis although most commonly in patients with advanced fibrosis and the lack of HCC screening in these patients partly explains the late diagnosis [5,6].

The mechanisms underlying the development of HCC in the context of NAFLD, especially in the absence of cirrhosis are not completely clarified and the identification of druggable biomarkers is crucial to improve its surveillance, diagnosis, and prognosis, as well as prevention. The present review aims to discuss the metabolic, genetic, dietary, and immunity-related factors which predispose to liver cancer in NAFLD patients, emphasizing the potential effect of nutritional therapy in HCC.

## 2. Common Genetic Variations Promote the Switch from NASH to HCC

Familial, twin, and epidemiological studies indicated that NAFLD has a strong heritable component. Both common and rare mutations contribute to NAFLD pathogenesis and to the transition from NASH to HCC [7,8]. The rs738409 C > G single nucleotide polymorphism (SNP) in the Patatin-like phospholipase domain containing 3 gene (*PNPLA3* or adiponutrin) is strongly associated with the entire spectrum of NAFLD, encompassing NASH, severe fibrosis and HCC [9,10]. *PNPLA3* gene codifies for a 481-aminoacid membrane lipase, located in the endoplasmic reticulum (ER) and on lipid droplets (LDs) surface in hepatocytes, adipocytes and hepatic stellate cells (HSCs) and the rs738409 variation codifies for an aminoacidic substitution from isoleucine to methionine at position 148 [11]. Patients who carry the at risk G allele lost PNPLA3 enzymatic activity, paralleled by reduced TG hydrolysis and dismissal thus leading to their accumulation in hepatocytes [12].

Although PNPLA3 is mainly involved in triacylglycerol remodeling, it may directly precipitate fibrogenesis and carcinogenesis, irrespective of steatosis by impairing retinol release from HSCs [13,14,15,16]. Indeed, the histological pattern of NAFLD patients carrying the PNPLA3 I148M variation was featured by macro and microvesicular steatosis, portal inflammation, high proliferation of hepatic progenitor cells (HPCs), ductular reaction, myofibroblast and HSCs activation, thus sustaining portal fibers deposition and systemic oxidative stress [17]. Furthermore, in NASH patients the expression of PNPLA3 significantly correlated with fibrosis stage and alpha-smooth muscle actin (α-SMA) levels thus suggesting that its metabolic regulation differs among hepatocytes and HSCs [18].

Finally, PNPLA3 exerts several effects on human liver metabolome influencing mitochondrial functions, glucose reprogramming and tumorigenesis [19]. Huh-7 hepatoma cells overexpressing the PNPLA3 I148M variant showed high levels of lactate and -glutamyl-amino acids, thus mirroring the metabolic switching towards aerobic glycolysis and mitochondrial failure, respectively [19]. In addition, hepatic overexpression of the I148M protein in mice promoted steatosis and NASH, by priming the metabolic reprogramming and the activation of inflammatory pathways driven by either increased triglyceride and ceramide species [20]. Intriguingly, Bruschi et al. demonstrated that HSCs overexpressing the I148M variation and exposed to transforming growth factor beta (TGF-β) strengthened aerobic glycolysis, as supported by high lactate release. Moreover, these cells showed activated Hedgehog and Yap pathways, mainly involved in the control of energy expenditure and maintenance of myofibroblastic traits [21]. Finally, it has recently demonstrated that HSCs from carriers of the homozygous PNPLA3 I148M variant were characterized by signatures of defective DNA repair, reduced TP53 signaling and oxidative stress, contributing to the development of hepatic carcinogenesis [22].

Later than PNPLA3, an exome-wide association study identified the rs58542926 C > T missense variant in the Transmembrane 6 superfamily member 2 (*TM6SF2*) gene as a determinant of hepatic triglyceride content, higher serum aminotransferases and lower levels of low-density lipoprotein (LDL)-cholesterol [23]. TM6SF2 localizes in the ER and ER-Golgi compartments, and it participates to hepatic very low-density lipoprotein (VLDL) lipidation and assembly in the ER cisternae. The rs58542926 variation, encoding a p.Glu 167Lys (E167K) aminoacidic substitution leads to a misfolded protein which is rapidly degraded in hepatocytes thus resulting in an impaired VLDL secretion and fat accumulation [23,24].

The *TM6SF2* minor T allele was also associated with lower serum cholesterol and triglyceride levels in several cohorts of NAFLD patients and in large population studies including the Dallas Heart Study, the Dallas Biobank and the Copenhagen Study [23,25]. In a large cross-sectional cohort of 1201 individuals with biopsy-proven NAFLD, we previously demonstrated that the E167K variation was associated with steatosis, inflammation, ballooning and fibrosis but it conferred protection against cardiovascular events [26].

In a multiethnic pediatric cohort including 957 individuals, the TM6SF2 E167K variation has been related to high hepatic fat content, high alanine aminotransferase levels, severe fibrosis and a more favorable lipid profile thus confirming its association with liver damage and protection against cardiovascular events in NAFLD patients [27].

Most of the data pointed at the role of TM6SF2 E167K variation in predisposing to all the NAFLD spectrum [26,28,29], although its impact on clinically relevant fibrosis and HCC is still controversial [29,30,31]. Liu et al. reported that the rs58542926 was associated with advanced hepatic fibrosis/cirrhosis in two histologically characterized cohorts encompassing steatosis, steatohepatitis, fibrosis and cirrhosis (combined n = 1074) regardless of other confounders as gender, sex, body mass index (BMI), T2D and *PNPLA3* rs738409 genotype [32]. The association between the rs58542926 variation, advanced fibrosis and HCC was furtherly described in a cross-sectional and in small cohort studies including 502 and 129 NAFLD patients, respectively although it had only a minor influence on hepatic fibrosis in viral hepatitis [29,33]. In a meta-analysis including a large pooled population made up of 24,147 individuals with heterogeneous chronic liver disorders, the E167K polymorphism was associated with NAFLD, higher risk of cirrhosis and HCC but not with viral hepatitis [34,35].

Finally, Longo et al. have recently demonstrated that *TM6SF2* silencing in HepG2 (*TM6SF2^−^*^/*−*^) hepatoma cells by clustered regularly interspaced short palindromic repeats/CRISPR-associated protein 9 (CRISPR/Cas9), resulted in an increased number of mitochondria with small and globular shape, loss of cistern architecture and ultrastructural electron density which may indicate mitochondrial failure and degeneration. Notably, the knock-out (KO) model when combined with membrane bound o-acyltransferase domain-containing 7 (*MBOAT7*) silencing runs into metabolic reprogramming towards anaerobic glycolysis, suggesting that the co-absence of *TM6SF2* and *MBOAT7* genes may synergically induce mitochondrial dysfunctions in hepatocytes thus contributing to the switch towards NASH up to HCC [36,37,38].

Following the time sequence, in 2015 a genome-wide association study (GWAS) which evaluated the genetic predictors of cirrhosis in alcoholics, identified the common rs641738 C > T variant in the *TMC4*/*MBOAT7* locus, as a novel inherited mediator of hepatic diseases [39,40]. MBOAT7, also known as lyso-phosphatidylinositol (Lyso-PI) acyl-transferase1 (LPIAT1, is a protein involved in the acyl chain remodeling of phospholipids via the Lands’ cycle. MBOAT7 is associated to the membranes bridging ER and mitochondria in which LDs and fat biosynthesis occurs and it is mainly expressed in hepatocytes, sinusoidal endothelial cells, immune cells and HSCs [41,42,43].

Mancina and Dongiovanni, demonstrated that the rs641738 variant predisposes to the NAFLD spectrum and the mechanism underlying this association relies on MBOAT7 reduced expression which leads to alteration in phosphatidylinositol (PI) species composition [43,44,45]. According to the impaired hepatic MBOAT7 function, patients carrying the T allele showed changes in plasma and hepatic PI species, decreasing specifically those enriched in omega-3 Polyunsaturated Fatty Acids (PUFAs) and increasing saturated ones [44,45]. This concept was recently reinforced by Meroni and colleagues who elegantly demonstrated that hepatic MBOAT7 down-regulation is a maladaptive response to hyperinsulinemia and that the impaired enzymatic activity forces hepatic fat storage in patients, in in vivo models, representative of NAFLD and in *MBOAT7* silenced HepG2 cells [43].

The rs641738 *MBOAT7* variation has also been related to progressive NAFLD and a possible mechanism which supports this association was proposed by Tanaka who demonstrated that MBOAT7 depletion in 3D-spheroids composed by hepatocytes and HSCs, stimulated the release of cytokines, fibrogenic markers and collagen deposition due to the accumulation of the MBOAT7 substrate Lyso-PI lipids [44,46,47]. Indeed, saturated Lyso-PI were higher in sera of patients affected by severe fibrosis compared to healthy subjects. Finally, we firstly demonstrated that the rs641738 T minor allele was associated with HCC in 765 Italian NAFLD patients, and more so in those without advanced fibrosis. These results were confirmed when we combined data from an independent UK NAFLD cohort (n = 913) and in a pooled population of non-cirrhotic patients with chronic hepatitis C or alcoholic liver disease (n = 1121) [41].

To sum up, the genetic variants which strongly predispose to HCC are those in *PNPLA3*, *TM6SF2* and *MBOAT7* genes which have been extensively described to promote hepatic fat accumulation and their effect is not necessarily mediated by the development of hepatic fibrosis [41,48]. Although the efficacy of these SNPs in predicting NAFLD-HCC is limited, it may be amplified by pulling them in polygenic risk scores (PRS) [49].

## 3. Genetic Variants in Immunoregulatory Genes Modulate the Risk of HCC in NAFLD Patients

In the last years, it is emerged that the immune response to fatty depots may influence NAFLD progression and the onset of HCC. Innate and adaptive immune cell activation together with oxidative stress, mitochondrial and ER dysfunctions lead to necro-inflammation and hepatocellular regeneration thus promoting HCC development [50]. It has been described that fatty liver modulates the immune microenvironment which is characterized by a lower number of anti-tumors CD4+ T cells and an increase of CD8+ T, natural killer and Th17 cells [51]. The remodeling of the immune cell population may impact on immunotherapy which has recently become a new therapeutic option for the management of HCC in terms of immune checkpoint blockers. Nivolumab and pembrolizumab, both monoclonal antibodies against programmed cell death protein 1 (PD-1) have been approved for treatment of HCC [52,53], although phase III trials failed to reach their primary endpoints to increase overall survival (OS) in patients with non-viral HCC [54].

The *PDCD-1* gene encodes an inhibitory cell surface receptor involved in the regulation of T cell functions during immune responses/tolerance. It binds to ligands PD-L1 and PD-L2 thus suppressing their activity and limiting potential damage to the host [55]. A sustained PD-1 expression, its decreased degradation, or expression of PD-L1 may also be observed in individuals susceptible to HCC development [55,56].

Pfister et al. reported that in preclinical models of NASH-induced HCC, immunotherapy against PD-1 increased the number of CD8+PD-1+ T cells within tumors but it did not lead to tumor regression thus suggesting that immune surveillance was impaired [50]. These authors conducted a meta-analysis of three large randomized controlled phase III trials of immunotherapy in patients with HCC from different etiologies and they found that OS was higher in subjects with viral-related HCC. Although these results did not differentiate between alcoholic liver disease and NAFLD/NASH, they were furtherly confirmed in a cohort of 130 patients with HCC in which NAFLD was associated with shortened survival after PD-1 therapy [50]. The poor response to immunotherapy in patients with non-viral HCC compared to viral ones may be due to different hepatic microenvironment or immune milieu, and these findings might also have implications for patients with obesity and NAFLD/NASH [50].

Polymorphisms in the *PDCD-1* gene have been associated with an increased risk of various types of cancers and some of them alter protein expression and function [55]. The *PDCD-1* rs10204525 C > T is located in the 3′ UTR, it increases PD-1 expression and has been associated with persistence in HBV infection [57]. The rs7421861 A > G in *PDCD-1* gene is localized in the intron 1, where there are several alternative splicing sites [24]. Both the rs10204525 and the rs7421861 increased the risk of esophageal cancer in Asian individuals and were associated with increased PD-1 expression. Furthermore, Kaplan-Meier survival curves showed higher *PDCD-1* gene expression contributed to worse survival of esophageal cancer patients [58].

In a cohort of 594 patients with NAFLD and 391 with NAFLD-HCC from three European centers, the *PDCD-1* rs7421861 was independently associated with NAFLD-HCC whereas the rs10204525 polymorphism reached significance after adjustment for confounding factors and more so in the smaller numbers of women with NAFLD-HCC. These associations were obtained in the UK cohort whereas the results were not confirmed in the Berna and Milan cohorts thus suggesting that genetic variants in genes which modify the HCC microenvironment may differ according to ethnicity although pathways may be shared [59].

## 4. The Pathogenic Role of Rare Genetic Variants in NAFLD-HCC Development

Rare genetic variants which strongly impair protein function thus exerting a pathogenic effect may contribute to fill the missing hereditability involved in NAFLD-HCC susceptibility. De Filippo et al. demonstrated that hepatomegaly, abnormal liver enzymes, steatosis, NASH and related complications were observed in patients with abetalipoproteinemia (ABL) and hypobetalipoproteinemia (Ho-FHBL) [60]. ABL is a rare autosomic recessive disease mainly caused by mutations in microsomal triglyceride transfer protein large subunit gene (*MTTP*), encoding for the Apolipoprotein B (ApoB) chaperon protein (MTP) thus leading to defects in chylomicrons and VLDL secretion. Ho-FHBL is a rare autosomal co-dominant disorder caused by mutation in ApoB100 and results in defects of b-lipoprotein secretion. Intra hepatic triglyceride content and higher incidence of NASH were found in patients under treatment with ApoB synthesis and MTP inhibitors [61].

Moreover, Ho-FHBL patients who had fibrosis were characterized by the co-presence of obesity and insulin resistance (IR), two conditions commonly related to NAFLD. It could be speculated that the higher predisposition to advanced liver damage in these patients may be due to the contribution of other mutations predisposing to severe fibrosis as *PNPLA3* [60]. Indeed, in a Caucasian father-son pair with NAFLD, obesity and low LDL cholesterol, both had a heterozygous mutation in *APOB* gene (c.1830-1G > A) which is a pathogenic splicing variant which causes truncated ApoB thus resulting in FHBL and they were heterozygous also for the *PNPLA3* rs738409 [62]. This father–son case series shows that clinically significant NAFLD phenotype may be the result of interacting effects of metabolic and disease-modifying genetic variants [62].

It has been recently demonstrated that patients with HCC related to NAFLD have an enrichment in rare pathogenic variants, in particular in *APOB* gene. Therefore, these mutations were collectively observed in a high proportion of Italian patients (15%), and pathogenic and truncating mutations in this gene were highly enriched in the overall cohort of NAFLD-HCC patients [63]. Notably, in line with a causal role of hepatocellular lipid retention due to a defect in VLDL lipidation in promoting NAFLD-HCC, somatic mutations in *APOB* gene also frequently occur during hepatic carcinogenesis [64].

In the attempt to decipher HCC molecular signature and to optimize personalized treatments, Kim et al. performed an exome sequencing analysis of NAFLD-HCC tumor samples and revealed that Telomerase reverse transcriptase (*TERT*) promoter mutations occurred in 82% of cases, followed by Catenin beta 1 (*CTNNB1*) (45%) and *TP53* (36%) mutations [65]. An Italian group evaluated the germline *TERT* mutations associated with NAFLD-HCC in 40 patients with NAFLD-HCC, 45 patients with NAFLD-cirrhosis, 64 healthy controls and examined telomere length. They detected an enrichment of *TERT* mutations in NAFLD-HCC and those with predicted functional impact co-segregated with liver disease in two families. Conversely, no mutations were found in cirrhosis and controls and telomere length was reduced in individuals with NAFLD-HCC versus those with cirrhosis and healthy controls [66].

The susceptibility to advanced fibrosis and carcinogenesis is also influenced by cellular senescence and cell cycle arrest. Therefore, the rs762623 in cyclin dependent kinase inhibitor 1A (*CDKI1A*) which encodes the cellular senescence marker p21, was significantly associated with the development of progressive liver disease in two cohorts of biopsy-proven NAFLD from UK (n = 323) and Finland (n = 123) [67].

We recently evaluated the impact of the rs599839 A > G variant, in the *CELSR2-PSRC1-SORT1* gene cluster, on liver disease severity in 1426 NAFLD patients of whom 131 had HCC. The frequency of the minor G allele was higher in NAFLD-HCC patients compared to those without cancer and it was associated with higher risk of HCC, independently of fibrosis severity, poor prognosis, and advanced tumor stage. Furthermore, hepatic PSRC1 expression was increased in NAFLD patients carrying the rs599839 variant and it was positively related to that of genes implicated in cell proliferation [68].

Furthermore, it has been demonstrated that the rs1800832 A > G variant in the 5′ UTR of the Neurotensin (*NTS*) gene associates with fibrosis, cirrhosis and HCC in 1166 NAFLD patients, likely by affecting NTS protein activity [69]. This variant synergizes with the rs6090453 polymorphism in the Neurotensin receptor 1 (*NTSR1*), further promoting severe liver damage in subjects carrying both the *NTS* and *NTSR1* at-risk alleles [69].

The mutational profiling of NASH-HCC tumors has been recently assessed by Pinyol et al. who collected 80 NASH-HCC and 125 NASH samples and performed expression array and whole exome sequencing. NASH-HCC tumors revealed *TERT* promoter (56%), *CTNNB1* (28%), *TP53* (18%) and Activin A Receptor Type 2A (*ACVR2A*) (10%) as the most frequently mutated genes. Moreover, the percentage of mutations in *ACVR2A* gene was higher in NAFLD-HCC compared to HCC from other etiologies and its in vitro silencing resulted in higher cellular proliferation rate. *ACVR2A* gene encodes for a cytokine receptor involved in cell differentiation and proliferation whose downregulation has been associated with poorer outcome in colorectal cancers thus suggesting it may act as tumor suppressor also in HCC [70]. Finally, the authors found that the tumor mutational burden was higher in non-cirrhotic NASH-HCC than in cirrhotic ones [22]. Intriguingly, NASH-HCC showed a unique tumor signature characterized by bile and fatty acid signaling, oxidative stress, inflammation, and mitochondrial dysfunction and in patients who carried the *PNPLA3* I148M variant it was enriched in defective pathways of DNA repair and reduced TP53 signaling, thus reinforcing the role of this polymorphism in HCC development.

## 5. Epigenetic Variations Driving NAFLD-HCC

The current knowledge supports the hypothesis that only less than 10% of NAFLD heritability may be justified by the above-mentioned genetic polymorphisms and the susceptibility to progress towards severe hepatic injuries might be explained by gene-environment interactions. The latter defines ‘epigenetics’, the reversible inherited phenomenon that may powerfully modify the expression of genes in response to environmental cues, without altering their DNA sequences [71]. Epigenetic remodeling includes DNA methylation, histone modifications and microRNA (miRNA)-targeting mRNA and the discovery of possible epigenetic modifiers constitutes a great opportunity to better outline reliable molecular indicators for the determination of early risk and of patients’ prognosis [71,72].

During the development of NAFLD, both nuclear DNA and mitochondrial DNA (mtDNA) are progressively affected by aberrancies in the process of DNA methylation, differentially describing disease stages [73]. In details, these aberrancies are mainly due to the activation of DNA methyltransferases (DNMTs), which are enzymes involved in the transfer of a methyl group from S-adenyl methionine (SAM) to the fifth carbon of a cytosine (5 mC) preceding a guanine nucleotide or CpG clusters. In particular, NASH patients are characterized by severely enhanced hepatic DNMT levels [74], whereby inducing a higher methylation pattern of specific genes, including the mitochondrially encoded NADH dehydrogenase 6 (MT-ND6) compared to those with simple steatosis [74]. Thus, it has been hypothesized that this epigenetic change in mtDNA may participate to the switching from simple steatosis to progressive NASH. These observations have been further corroborated by Kuramoto et al. who determined that NASH-related tissues had a specific DNA methylation motif, that possibly intervene in the process of hepatocarcinogenesis by favoring the silencing of genes implicated in the repair of damaged DNA and in apoptosis [75]. In keeping with this notion, dietary deficiency of methyl group donors, such as choline, betaine, vitamin B12 and folate boosts epigenetic anomalies favoring in turn, advanced liver damage and neoplastic transformation. Indeed, in rodents a methyl-deficient diet provides stable alterations in DNA methylation promoting carcinogenesis [76].

Alongside, variations in DNA packaging due to post-translational histone modifications may be dependent of environmental *stimuli*. For instance, the histone deacetylase 8 (HDAC8) has been defined as a modifier of chromatin organization in NASH-related HCC in rodents and in humans, given its oncogenic properties. In dietary models of NASH and HCC, the expression of HDAC8 is regulated by Sterol Regulatory Element Binding Transcription Factor 1 (SREBP1) and exerts its function physically interacting with polycomb protein enhancer of zeste homolog 2 (EZH2) to force aberrant cell proliferation. Indeed, both in rodents and in patients with NAFLD-HCC, the activation of HDAC8/EZH2 complex inhibits p53/p21-mediated apoptosis, cell-cycle arrest, and stimulates β-catenin-dependent cell proliferation, whereby controlling histone H4 deacetylation and H3 lysine 27 trimethylation. Thus, it works as epigenetic silencing machinery on inhibitors of Wingless-related integration site (Wnt)/β-catenin signaling and favors HCC development [77]. In addition, a global perturbation of histone H4K16 acetylation, favoring in turn its deacetylation, has been observed in Stelic Animal Model mice, a rodent model of human NASH-related HCC [78]. The persistent deacetylation of genes implicated in cell death pathways facilitated their silencing contributing to the NASH-derived HCC onset [78].

Finally, ever-increasing evidence supports the role of miRNAs in the epigenetic deregulation of metabolic processes in NAFLD, NASH and HCC [79]. We have previously extensively discussed the hepatic and circulating miRNA signature related to all hallmarks of NAFLD, up to NASH and HCC [11,71,80]. For example, the reduction of miR-122 has been pointed out as a direct inducer of NASH-associated HCC [81]. Moreover, miR-15/16 cluster exerts a tumor suppressor role, inhibiting various oncogenes and cell proliferation [82,83]. Hence, its expression is restrained in highly invasive HCC cell lines, in aggressive HCCs with lymph nodes metastasis and elevated TNM classification [82,84]. Consistently, it has been shown that the expression of miR-34a is shortened in hepatoma cells as well as in tumor samples, since it exerts its anti-malignancy activities via p53/miR-34a/SIRT1 positive feedback loop [85,86]. An opposite effect on tumorigenesis is mediated by miR-221. Indeed, its over-expression favors cell growth and invasion in cultured cells, and it correlates with poor prognosis and with sorafenib resistance in HCC patients [87,88,89]. Several studies reported deregulated miRNAs in cancerous tissues compared to non-tumoral ones albeit these findings are conflicting, possibly due to different technical approaches, disease etiology, genetic background, and many other biases.

## 6. Inflammation

Hepatic IR and obesity are both well-established conditions that induce systemic changes, including alteration of immune functions and favor a chronic low-grade inflammation [90]. These events may prompt a pro-inflammatory microenvironment, determining a higher risk to develop NASH and creating a clinical condition more prone to HCC onset [91,92].

Indeed, during NASH a sterile inflammation occurs, since damage-associated molecular patterns (DAMPs) released from damaged cells may trigger inflammasome response, leading to the maturation and secretion of both interleukin (IL)-1 and IL-8 sustaining inflammation [93]. DAMPs receptors belonging to the Toll-like receptors family (TLRs) are localized on the surface of Kupffer cells, HSCs, cholangiocytes and on endothelial cells (LSECs), emphasizing the immune response, the hepatic damage, and the extracellular matrix deposition.

Noteworthy, excessive reactive oxygen species (ROS) production due to the enhanced fatty acids beta-oxidation disrupts the respiratory chain, leading to mitochondrial defects and cytochrome-c discharge [94]. In addition, it has been demonstrated that ROS species promote inflammatory cytokines production such as tumor necrosis factor-alpha (TNF-α), IL-6 and leptin thus perpetuating the inflammatory cascade and recruiting circulating monocytes and lymphocytes [95]. TNF-α and IL-6 in turn may also activate the pro-oncogenic c-Jun N-terminal kinase (c-Jun) and Signal Transducer and Activator of Transcription 3 (STAT3), respectively whereas leptin exerts a profibrotic and carcinogenic role by upregulating TERT expression [96]. Moreover, IR and radicals of oxygen may activate per se nuclear factor kappa-light-chain-enhancer of activated B-cells (NF-κB) signaling pathway, thus amplifying inflammation mainly through IL-6, and promoting STAT3-mediated cell survival [97].

The unfolded protein response (UPR) and calcium extrusion from ER stores, have been frequently observed in NASH patients. Excessive calcium amount forces mitochondrial permeabilization, further enhancing ROS production and caspases activation [98]. When reactive oxygen products exceed the capacity of the protective enzymes, glutathione peroxidase and catalase, the exaggerated oxidative stress causes lipid peroxidation, genomic instability, apoptotic death, and pro-inflammatory mediator secretion from injured hepatocytes, creating a context which strongly promotes HCC development.

## 7. Gut Microbiota

As a consequence of the tight anatomo-functional crosstalk between gut and liver, the gut-liver axis may exert several implications in the development of progressive NAFLD towards HCC [99]. The liver is constantly exposed to a flow of potentially dangerous microbial by-products and nutrients, derived from the gut through the venous system of the portal circulation. In turn, the liver may modulate the microbiota composition by the bile acids secreted into the duodenum lumen [99]. Gut microbiome facilitates the host defense against harmful pathogens, influencing at local and systemic level both the innate and adaptive immune response. Notwithstanding, mucus erosion, reduction of antimicrobial peptides (i.e., defensins, lysozyme, and c-lectin Reg3b/g) and Immunoglobulin A (IgA), have been associated with enhanced gut permeability, translocation of pathogenic microorganisms and gut-derived toxins (endotoxemia) whereby establishing a chronic low-grade inflammatory state as reported in preclinical and human studies [100,101,102,103]. Alterations in the barrier integrity (*leaky gut)* together with the disproportion in gut microbiota composition frequently occur in patients affected by severe NAFLD [104,105]. Specifically, the definition ‘dysbiosis’ points out to all quantitative and qualitative variations that may imbalance the taxonomic composition of beneficial and pathogen bacteria [106]. This variability may be dependent on age, lifestyle, medications and diets [107,108,109,110]. For instance, the consumption of a Western diet may favor intestinal bacterial overgrowth, endotoxins translocation, mucosal inflammation, and immune system activation. Therefore, the phenomenon of dysbiosis along with disturbances in the gut-liver axis may define the transition of steatosis up to NASH and HCC [111,112,113,114,115].

In this context, dysbiotic flora favoring *Escherichia coli* expansion results into the increase of endogenous molecules such as ethanol, ammonia and acetaldehyde, activating in turn hepatic Kupffer cells to produce pro-inflammatory cytokines [99,116]. In addition, several pathogen-associated molecular patterns (PAMPs) among which lipopolysaccharides (LPS) and peptidoglycans prime the activation of Toll-like receptors (TLRs) on hepatocytes, Kupffer cells and HSCs, precipitating systemic inflammation and fibrosis [117,118]. Likewise, DAMPs may perpetuate the inflammation via intracellular nucleotide-binding and oligomerization domain (NOD)-like receptors (NLRs) activated by TLRs (e.g., TLR2, TLR5) and inflammasome, which enhances interleukins production in hepatocytes, Kupffer cells and HSCs [119].

Imbalances in gut microflora communities contribute to severe hepatic inflammation. Specifically, an enrichment in *Cytophaga–Flavobacter–Bacteroides* phyla favors IL7 secretion from T-helper cells (Th17) [120] and an elevated abundance of *Bacteroides* and *Ruminococcus* have been independently associated with NASH and fibrosis [121]. These abnormalities have been further corroborated by exploring the fecal bacterial ratio between *Bacterioidetes* and *Firmicutes* in pediatric NAFLD patients, in which the abundance of *Bacterioidetes* is enhanced, while the levels of *Firmicutes* are shortened [116].

Notably, intestinal flora anomalies may be causally implicated in the transition to HCC [122]. A peculiar cancerous fecal microbiota enriched in the phylum *Actinobacteria* and in 13 genera, including *Gemmiger* and *Parabacteroides* distinguishes HCC from cirrhotic patients [123]. Specifically, endotoxin-producing genera were increased early in fecal samples from HCC patients, whereas the beneficial butyrate-producing ones decreased [123]. Notwithstanding, Yu and colleagues reported that host microflora sterilization represses tumor onset, strikingly dampening the number and dimension of nodules in diethyl nitrosamine (DEN)-induced HCC rodent models [124]. According to these observations, the administration of LPS to mice grown in germ-free conditions reverted this situation [125]. Furthermore, LPS/TLR4 signaling pathway may promote hepatocarcinogenesis by favoring the senescence-associated secretory phenotype (SASP) in activated HSCs and the secretion of chemoattractant cytokines and of tumor-promoting factors, as well as damaged DNA [126,127]. These findings support the notion that gut microflora and TLR4-mediated inflammation are required for tumorigenesis [124,125].

## 8. Nutrition and HCC

A broad number of metabolic and environmental modifiers, such as lifestyle and food choices may contribute to the development of NASH-related HCC [51]. Dietary habits and diet composition, in terms of macro and micronutrients, have been found to be modulators of chronic diseases prognosis. Indeed, the pathogenesis and the aggressiveness of NASH-driven HCC are convoluted, and they entail intricate routes, encompassing immune response, oxidative stress, autophagy, organelle derangement and DNA damage [51]. All these events may be partially influenced by alimentary and behavioral attitude. Therefore, nutritional interventions aimed to ameliorate the metabolic status of patients may be helpful to counteract to NASH progression to cirrhosis and HCC, and to maximize benefits by combining drug-diet approaches.

Several clinical trials are focused on nutritional interventions in patients with chronic liver injuries, with the purpose to reduce HCC incidence and improve the quality of life of patients. However, the precise effect of each eating pattern on hepatocarcinogenesis is still under definition. A case-control study conducted in 641 cases and 1002 controls, demonstrated that a vegetable-based dietary pattern protects against HCC risk, whereas Western diet (WD) correlates with enhanced liver carcinogenesis [128]. These observations have been supported by a meta-analysis across 19 studies involving 1,290,045 participants and 3912 cases of HCC, that demonstrated that a diet enriched in vegetables, but not in fruits, reduces the HCC risk [129]. According to these findings, a close adherence to Mediterranean diet associates with lower risk of HCC, with an odd ratio (OR) 0.51 (95% CI, 0.34–0.75) [130]. Likewise, in the European Prospective Investigation into Cancer and Nutrition (EPIC) cohort, it has been investigated the correlation between lipid consumption, lipid assumed subtypes and fat sources with HCC incidence, showing an inverse association between total fat intake and HCC risk, which was primarily due to monounsaturated lipids ingestion rather than polyunsaturated ones [131]. In the same cohort, it has been reported that total fish assumption defended against liver carcinoma [132]. As well as dietary fibers down-modulate the susceptibility to develop HCC [133]. Conversely, the impact of meat consumption in HCC onset is still debate [134]. Hence, the assessment of nutritional status in patients with NASH, characterized by an increased predisposition to HCC may assume a relevant prognostic purpose and international recommendations may be necessary to support current therapies, with the pursuit to improve liver function and prolong survival.

### 8.1. Alcohol Drinking Accelerates NASH-HCC Onset

Alcohol intake has been widely reported to be associated with an increased risk of HCC [135]. Indeed, around 10–20% of alcohol abusers develop hepatic cirrhosis, and 1.9–2.6% of them HCC [136]. This proportion seems to be directly dependent not only on the amount of alcohol consumed, but also on concomitant metabolic and genetic risk factors [137]. For instance, diabetes and obesity have a synergistic interaction with alcohol consumption, further enhancing the susceptibility to HCC [138,139]. Similarly, in patients who already suffer from NASH, ethanol consumption hesitates in a 3.6-fold higher risk of HCC compared to those patients without lifetime alcohol consumption [140]. Thus, ethanol intake may constitute a modifiable risk factor for HCC even in quantities generally considered safe. Notably, even in children affected by NASH, dysbiosis enhances blood ethanol levels, because of the intensification of carbohydrate catabolism by alcohol-producing bacteria, such as *Escherichia coli* [116,119].

The mechanisms whereby alcohol misuse induces HCC onset seems to be related to alcohol-mediated inflammation, oxidative stress, genotoxicity, DNA methylation alterations and DNA instability predisposing to strand breaks and chromosomal loss [141]. However, the precise events that precipitate liver damage up to HCC are not completely understood.

Firstly, ethanol is metabolized to acetaldehyde by the Alcohol Dehydrogenase (ADH). Acetaldehyde is a highly reactive and toxic compound, that can create adducts with macromolecules (i.e., proteins, DNA, or lipids) thus impairing their function. Then acetaldehyde is oxidized to acetate by Aldehyde Dehydrogenase (ALDH) in mitochondria. These two reactions reduce NAD+/NADH ratio, favoring NADH re-oxidation to NAD+ in the mitochondria, fat accumulation and generating ROS [142]. Likewise, even the Cytochrome P450 2E1 (CYP2E1), induced by alcohol consumption triggers the activation of de novo lipogenesis, oxidative stress, lipid peroxidation and inflammation [143]. As a consequence, the activation of inflammatory cells in the context of steatohepatitis, may prompt the release of inflammatory cytokines and chemokines, favoring the transition of HSCs to myofibroblasts [143]. Therefore, steatohepatitis is a rate limiting step for the development of advanced liver injuries, among which cirrhosis and HCC. Acetaldehyde per se exerts a direct pro-carcinogenic effect, while CYP2E1 metabolizes pro-carcinogenic compounds which are present in alcoholic drinks. Finally, higher levels of LPS in alcohol consumers promote cancer stem cells proliferation [99,144].

### 8.2. The Role of Aflatoxin B1 in Hepatocarcinogenesis

Aflatoxin B1 (AF-B1), a secondary fungal by-product derived from *Aspergillus*, is a frequent contaminant of grain, milk, rice, cereals and maize, vegetables, and nuts [145]. AF-B1 has potent genotoxic and carcinogenic effects, likely by inducing point mutations in the *TP53* gene and its chronic exposure fosters the suppression of acute inflammatory response, favoring in turn HCC spreading [146]. Thus, it represents the most important dietary-derived compound that increases the susceptibility to develop HCC. Its carcinogenic potency is exacerbated by the co-presence of hepatitis B infection (HBV), synergistically enhancing the risk of HCC [147]. However, limitations of the consumption of these potentially harmful products are suggested even independently of HBV. To date, no specific dietary recommendation is available for patients affected by NASH and NASH-related cirrhosis, who have per se a 7-fold higher risk to develop HCC compared to matched controls [148]. As well as, in the case of alcohol over-consumption, LPS-triggered inflammation may further increase the AF-B1 hepatotoxicity in rodents [149,150]. In addition, AF-B1 may derange intestinal barrier function [151]. The presence of urinary aflatoxin-N7-guanine and aflatoxin-serum albumin adducts have been studied as biomarkers and their modulation by various agents has been proposed in clinical trials as surrogate outcomes of the chemo-preventive efficacy [152]. For instance, broccoli sprout extracts decrease urinary excretion of sulforaphane metabolism and aflatoxin-DNA adducts [153]. Moreover, Curcumin and Resveratrol by exerting anti-inflammatory and anti-apoptotic effects, improve the aflatoxin-induced hepatocarcinogenesis [154,155].

### 8.3. Iron Overload Increases the Risk of HCC

Later manifestations of iron overload include cirrhosis and cirrhosis-related HCC in patients with hereditary hemochromatosis or chronic hepatic inflammation [156]. Phlebotomy and chelating agents may dampen the risk of HCC in patients with siderosis. Indeed, iron-depots are frequent even in patients with NASH and more so in those with NASH-driven HCC [157]. Iron deposits induce the formation of highly reactive hydroxyl radicals, which may mediate mitochondrial damage and precipitate NASH into cirrhosis and HCC [158]. Dietary iron restriction in mice models of NASH hampers oxidative stress, inflammation and fibrosis, due to a reduction of hepatic iron levels [159]. These findings suggest that a low-iron diet may provide beneficial effects not only in patients affected by severe hemochromatosis but also in those with NASH with the aim to prevent its progression towards more severe damage.

A similar mechanism has been observed for diets enriched in glucose, that may promote neoplastic transformation, by inducing the advanced glycosylation end product-specific receptor (AGER), that stabilize the oncoprotein c-Jun via O-GlcNAcylation thus supporting cell proliferation [160].

### 8.4. Dietary Cholesterol: The Main Lipid Driver of the Switching from Simple Steatosis to NASH-HCC

A growing body of evidence indicates that dietary cholesterol may represent an independent risk factor for HCC. Indeed, clinical and preclinical studies highlighted an association between cholesterol intake and the raising of NASH-related HCC, even in the absence of cirrhosis [161,162,163]. In obese and diabetic mice, cholesterol overload leads to lipotoxic accumulation of free cholesterol into the hepatocytes, attributable to the induction of genes related to cholesterol synthesis as SREBP2, to the suppression of cholesterol conversion into bile acids and their secretion [161]. Cholesterol accumulation in ER lumen prompts ER membranes disruption, causes the inhibition of sarco/ER calcium ATPase (SERCA) activity, exasperates oxidative stress, mitochondrial dysfunction, ATP depletion, lipotoxicity and hepatocyte degeneration, priming the activation of inflammatory cells and prompting the transition from simple steatosis towards NASH and fibrosis [161,164,165]. Furthermore, by adding to cholesterol a high fat challenge, the development of IR accelerates NASH and oxidative stress, aggravating liver inflammation [163]. Cholesterol overload seems to be able to foster Kupffer cells and HSCs activation [166]. In the former the internalization of cholesterol is mediated by the scavenger receptor (SR-A) or by CD36, leading to pro-inflammatory cytokine release, whereas in HSCs cholesterol uptake is performed by lectin-like oxidized LDL receptor-1 (LOX-1). The persistence of all these triggers promote the release of oxidized mtDNA, tumor growth and tumor reprogramming [164,165]. However, the precise event cascade through which cholesterol induces NASH-related HCC is still unclear.

In keeping with its pro-carcinogenic role, free cholesterol is severely accumulated in NASH patients, as a consequence of the imbalance between its biosynthesis, conversion and excretion and the formation of its depots correlates with hepatocyte degeneration and fibrosis [167,168]. Consistently, cholesterol consumption has been associated with a higher incidence of HCC in a population-based study among 14,407 participants [162]. In addition, serum cholesterol levels are positively correlated with growth, invasion and aggressiveness of carcinoma in patients with HCC [169]. Collectively, these observations point out free cholesterol accumulation as a common risk factor that drives both NASH and HCC development.

Liang and colleagues established that mice fed high fat high cholesterol (HFHC) diet treated with DEN displayed NASH development accompanied by more numerous and large liver tumors compared to animals treated with DEN and fed HFD alone. In addition, tumor specimens isolated from these mice are characterized by a specific aberrant gene expression pattern of cancer-related and metabolism-related genes, and by a more pronounced amount of non-synonymous somatic mutations due to the oxidative DNA damage and inflammation [170].

Notably, cholesterol-induced NAFLD–HCC generation is associated with gut microbiota dysbiosis and microbiota transplantation from HFHC mice to germ-free mice induces hepatic steatosis, inflammation, and cell proliferation. Conversely, atorvastatin administration, a drug used in the treatment of hypercholesterolemia, restores intestinal dysbiosis preventing HCC [171]. Hence, statins, widely used as lowering plasma cholesterol agents, seem to have a protective effect on HCC risk (hazard ratio HR, 0.48; 95% CI, 0.24–0.94) although further studies are required to confirm this association [172].

Finally, in absence of elevated dietary cholesterol levels, cancerous cells may upregulate endogenous cholesterol biosynthesis and cholesterol utilization with the purpose to maintain high cell proliferation, cell membranes neo-synthesis and to compensate metabolic demands [173]. Furthermore, cholesterol metabolites, such as 27-hydroxycholesterol and 6-oxocholestan-3beta,5alpha-diol, display tumor-promoter properties and accelerate hepatocarcinogenesis [174].

### 8.5. Protective Compounds against Hepatic Damage

Coffee consumption was often associated with benefits for a variety of diseases including metabolic syndrome, cardiovascular disease and chronic liver diseases [175]. In particular, a very recent study performed in 494,585 subjects from the UK Biobank cohort clearly indicated that all types of coffee are protective against hepatic steatosis (HR, 0.80, 95% CI 0.75–0.86) and HCC (HR 0.80, 95% CI 0.54–1.19) [176]. This beneficial effect of coffee is dose dependent, declining the risk of HCC of about 43% in individuals who usually consumed coffee [177]. Indeed, caffeine ameliorates cell proliferation, exerting antioxidant and anti-neoplastic properties through its compounds such as diterpenes, cafestol and kahweol, which modulate phase 2 hepatic enzymes involved in carcinogen detoxification and excretion [178]. Likewise, regular use of tea is enabled to produce similar hepatoprotective benefits, improving oxidative DNA damage [179]. Similarly, other dietary antioxidant agents such as coenzyme Q (12), vitamin C and E, selenium, phytochemicals (e.g., ellagic acid, curcumin, lycopene, epigallocatechin gallate, and resveratrol) enriched in fruit, vegetables, herbs and medicinal plants may have a protective role against hepatocarcinogenesis [180,181].

Superimposable results have been obtained by investigating the relationship between circulating Vitamin D and the risk of HCC. Vitamin D is a lipophilic hormone that is involved in calcium homeostasis, by promoting bone mineralization and remodeling, since it stimulates calcium and phosphorus absorption in the gastrointestinal trait [182]. In addition, it may play a key role in inflammation and cell differentiation [183]. A meta-analysis across 11 studies indicated that Vitamin D deficiency almost doubled the risk to develop HCC. In details, the reduction of Vitamin D significantly amplifies the HCC risk compared to healthy individuals (relative risk (RR) 2.16, 95% CI 1.20–3.88), irrespectively of the ethnicity of patients enrolled [184].

### 8.6. Dietary Fibers 

Individuals consuming a high-fibers diet (enriched in cereals, legumes, fruits, and vegetables) severely differ in gut microflora taxonomic composition compared to those who prefer WD, favoring the predominance of species which metabolize dietary plant polysaccharides [185,186]. The fermentation of soluble fibers mainly by intestinal bacteria belonging to the phyla *Firmicutes*, generates short-chain fatty acids (SCFAs), i.e., acetate, butyrate and propionate [187]. This process provides energy supply to mucosal cells of the host, benefits for health and favors the intestinal barrier integrity preservation and immune tolerance guaranteeing the eubiosis. Furthermore, a plant-based diet reduces fecal pH, due to the products of gut fermentative metabolism and to the hampered growth of pathogens along with *Escherichia Coli* and *Enterobacteriaceae* [188,189]. A reduction in butyric acid-producing bacteria weakens the connections between intestinal epithelial cells, by decreasing the expression of the tight junction proteins and mucins. In turn, the restoration of physiological abundance of microorganisms-producing butyrate, ameliorate the gut high permeability and systemic inflammation [190].

Alongside, mounting evidence indicates that SCFAs, mainly butyrate, play relevant immunomodulatory functions [191], regulating T-cell immunity [192,193,194]. For instance, SCFAs may mediate immune response and anti-inflammatory cytokine secretion (i.e., IL-10 and IL-12) [193], modulate size and function of the colonic CD4+CD25+ regulatory T cells (Treg) pool [192], promoting their activation at the expense of T helper (Th) 17 cells [194] and suppressing inflammation and protecting against cancer [195]. However, conflicting results have been recently obtained, showing in contrast that the exaggerate elevation of SCFAs in a context of dysbiosis may create a tumor-promoting microenvironment [196]. Hence, it has been assumed that the impact of SCFAs is strikingly dependent on the context, in terms of cell type, concentrations and time of exposure.

Even more, the composition of gut microbiota and its by-products among which the SCFAs may be responsible of epigenetic changes, affecting global histone acetylation and methylation in host tissues in a diet-dependent manner. In particular, mice fed a diet containing low levels of fermentable complex polysaccharides, displayed loss of cecal SCFA production, hesitating into a profound post-translational modification of hepatic histones, such as lower methylation of H3 histones in specific aminoacidic position (H3K27me1 and H3K36me2) [197,198]. Conversely, SCFAs may reduce the risk of carcinogenesis whereby inhibiting cell proliferation and invasion, suppressing HDACs, and inducing apoptosis [199,200].

### 8.7. Branched-Chain Amino Acids

Leucine, isoleucine and valine, also known as branched-chain amino acids (BCAA), are three essential amino acids, that are involved in various biological processes [201]. In patients with advanced liver damages and cirrhosis, plasma concentration of BCAA declines, due to nutritional disturbances. In rodents, BCAA administration is enabled to suppress DEN-induced liver tumorigenesis [202]. Similarly, BCAA supplementation in mice fed an atherogenic and high-fat (Ath+HF) diet, that induces NASH, refines the entire histological spectrum of liver damage and tumor incidence [203]. In particular, BCAA-enriched diets may alleviate steatosis and oxidative stress, upregulating the expression of the master regulator of mitochondrial biogenesis, the peroxisome proliferator-activated receptor γ coactivator-1α (PGC-1α) [204]. Similarly, BCAA consumption in patients alleviates the risk of liver-related complications along with encephalopathies, ameliorates the prognosis and improves serum albumin concentrations, restoring the nutritional status. Besides, prospective clinical trials demonstrated that they may reduce the occurrence of HCC in patients with cirrhosis [205,206].

### 8.8. Omega-3 Polyunsaturated Fatty Acids

Fish is the main source of Omega-3 PUFAs. It has been reported that its consumption reduced the risk of HCC by 35%, in a dose-dependent manner as a consequence of the enhanced dietary intake of Omega-3 PUFAs [207]. Indeed, PUFAs inhibit HCC growth through simultaneously inhibition of COX-2 and β-catenin [208]. In addition, diets enriched in PUFAs ameliorate mitochondrial fat oxidation, reduce abdominal circumference, plasma cholesterol and hepatic lipid synthesis [209,210]. The mechanism through which PUFAs may reduce the susceptibility to develop liver cancer, may be related to their ability to hamper NF-kB activation, cytokine secretion and oxidative stress by activating the peroxisome proliferator activating receptors (PPARs) [211].

**Figure 1:**
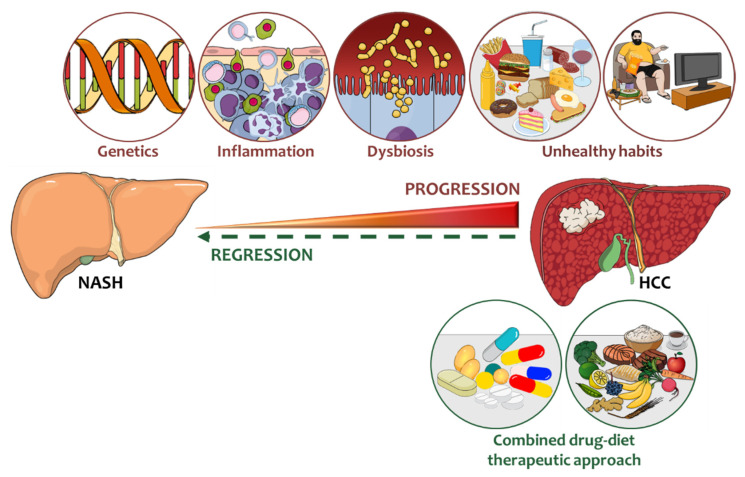
Risk factors that may intervene in the switching from NASH to HCC and current therapeutic approaches. Common and rare genetic variations that influence hepatic lipid handling, immune response and cell proliferation are strongly entangled in the transition towards liver cancer. Likewise, epigenetic phenomena including DNA methylation and histone modifications as well as miRNA perturbations may participate to these deleterious processes. Together with genetics even environmental risk factors such as unhealthy dietary habits, sedentary lifestyle, intestinal high permeability may further exacerbate liver inflammation, ER and oxidative stress, triggering the evolution to HCC. In this regard, nutritional and lifestyle interventions aimed to restore healthy behavior of patients may be helpful to counteract to NASH progression to cirrhosis and HCC. Notably, the combination of current therapeutic strategies (tumor ablation, pan-tyrosine kinase inhibitors, checkpoint blockade and immunotherapy) with dietary advice may maximize benefits, with the pursuit to improve liver function and prolong survival.

Risk factors that may intervene in the switching from NASH to HCC and possible combination between current therapeutic approaches and diet are summarized in Figure 1.

## 9. Preclinical Models to Induce NASH-HCC: From Dietary Supplementation to Genetics

As mentioned before, dietary composition may strongly impact on the development of NASH-derived HCC. However, few preclinical models that may recapitulate the entire spectrum of NAFLD until HCC are available to date. Mice fed high fat (HFD) or western (WD) diets slowly progress to HCC or do not develop HCC at all. An escape to this trouble has been proposed by Dowman et al. who showed that the American Lifestyle-Induced Obesity Syndrome (ALIOS) model, consisting in an administration of corn syrup enriched in trans-fats and fructose coupled with a sedentary lifestyle, may promote NASH and HCC onset after 12 months in only 60% of animals [212]. Conversely, in C3H/He mice, an ALIOS diet challenge induces macroscopic tumors, carrying a transcriptional landscape similar to human HCC, in 96% of animals at 48 weeks of age [90].

Similarly, a long-term feeding of a choline-deficient high-fat diet (CD-HFD) induced the activation of inflammatory pathways comparable to NASH patients. In this context, the inflammatory microenvironment encompassing the activation of CD8(+) and NKT cells, prompted NASH-to-HCC transition in about 25% of mice after 12 months [213]. Hence, due to the long-term exposure needed to develop advanced hepatic injuries, it is often preferred to combine a nutritional strategy with toxic compounds to boost hepatocellular neoplasms in mice. The most exploited chemical carcinogen to promote liver nodules formation is DEN, which may be associated with HFD or CD-HFD [95,214]. In these models, tumors onset seems to be dependent of the secretion of tumor-promoting inflammatory cytokines, among which IL-6 and TNFα, which activate in turn the oncogenic transcription factor STAT3 [95]. Similarly, even intraperitoneal carbon tetrachloride (CCl_4_) injections accelerate extensive fibrosis and HCC in mice fed a WD, resulting in histological, immunological and transcriptomic features close to human NASH-HCC in 24 weeks [215]. Likewise, the administration of low doses of streptozotocin (STZ), a DNA-damaging alkylating agent, immediately after birth, followed by HFD (STAM model) may be exploited to induce adenomas and HCC, at 12 and 16 weeks respectively [216].

Other examples of murine models that offer the possibility to reproduce NASH and HCC are the genetic ones. Among them, a diet-induced animal model of non-alcoholic fatty liver disease (DIAMOND) obtained by a cross of two common mouse strains, 129S1/SvImJ and C57BL/6J, fed for at most 52 weeks a high fat diet accompanied by high fructose and glucose subsequentially promotes all features of NAFLD up to HCC [217]. Alongside, MUP-uPA mice, transgenic rodents who overexpress urokinase plasminogen activator (uPA), are more prone to liver carcinoma onset upon a HFD, as a result of immune infiltration and of hepatocyte ER stress, which enhances lipogenesis [218]. Other genetically induced mice models of NASH-driven HCC may constitute an attractive opportunity to deeply understand the molecular mechanisms underlying tumorigenesis, i.e., hepatic specific phosphatase and tensin homolog (PTEN) KO mice (*AlbCrePten^flox^*^/*flox*^) [219] or liver specific STAT5/glucocorticoid receptor (GR) null mice [220] or mice lacking the methionine adenosyltransferase (MAT) gene (MATO mice) hesitating in a chronic reduction in hepatic S-adenosylmethionine levels [221] or melanocortin 4 receptor-deficient mice (MC4R-KO) fed HFD [222].

Ultimately, it has been recently demonstrated that mice carrying a loss-of-function mutation in the *Alms1* gene, also known as *Foz*/*Foz* mice, display hyperphagia and multiple aspects of metabolic syndrome, among which obesity, IR, dyslipidemia and hypertension [223,224]. In addition, when *Foz*/*Foz* mice are fed with a WD rapidly develop NASH in 4 weeks and advanced fibrosis in 12 weeks of diet, mimicking human pathobiology. After 24 weeks of WD, the 75% of *Foz*/*Foz* mice show the signs of cirrhosis and of hepatocellular malignancy [224]. Thus, this model may more faithfully resemble human disease etiology of NASH-HCC in a short time frame [223].

## 10. Concluding Remarks

The proportion of HCC attributed to NASH has been rapidly increasing in Western countries, and in 20–30% of cases hepatic tumor development may occur even in the absence of cirrhosis [225]. Thus, there is an urgent need to implement surveillance programs, focusing not only on patients with advanced fibrosis.

The pathogenesis of NASH-related HCC is complex and encompasses genetic and environmental risk factors, immune response, oxidative stress, organelle derangement and DNA damage. All these events may be partially influenced by alimentary and behavioral attitude. In this context, nutritional interventions and the combination of genetic variants in PRS may be helpful to predict and counteract NASH progression to cirrhosis and HCC thus maximizing the benefits of current therapies.

A novel frontier in the management of NASH-HCC is represented by the manipulation of the immune system through chimeric antigen receptor (CAR) T cells, vaccination using peptides or DNA, cytokine/chemokine antibody blockade, adoptive immune cell transfer and monoclonal antibody against PD-1 although large clinical trials are required to confirm their efficacy.

## Data Availability

Not applicable.

## Abbreviations

α-SMA	alpha-smooth muscle actin
ABL	abetalipoproteinemia
ACVR2A	Activin A Receptor Type 2A
ADH	Alcohol Dehydrogenase
AF-B1	Aflatoxin B1
AGER	advanced glycosylation end product-specific receptor
ALDH	Aldehyde Dehydrogenase
ALIOS	American Lifestyle-Induced Obesity Syndrome
APOB	apolipoprotein B
Ath+HF	atherogenic and high-fat
BCAA	Branched-Chain Amino Acids
BMI	body mass index
CAR	chimeric antigen receptor
CD-HFD	choline-deficient high-fat diet
CDKI1A	cyclin dependent kinase inhibitor 1A
c-Jun	c-Jun N-terminal kinase
CRISPR/Cas9	clustered regularly interspaced short palindromic repeats/CRISPR-associated protein 9
CTNNB1	Catenin beta 1
CYP2E1	Cytochrome P450 2E1
DAMPs	damage-associated molecular patterns
DEN	diethylnitrosamine
DIAMOND	diet-induced animal model of non-alcoholic fatty liver disease
DNMT	DNA methyltransferases
EPIC	European Prospective Investigation into Cancer and Nutrition
ER	endoplasmic reticulum
EZH2	polycomb protein enhancer of zeste homolog 2
FFAs	free fatty acids
GWAS	genome-wide association study
HBV	hepatitis B infection
HCC	hepatocellular carcinoma
HDAC8	histone deacetylase 8
HFD	high-fat diet
HFHC	high fat high cholesterol
Ho-FHBL	hypobetalipoproteinemia
HPCs	hepatic progenitor cells
HR	hazard ratio
HSCs	hepatic stellate cells
HSD17B13	hydroxysteroid 17-β dehydrogenase 13
ILs	interleukins
IgA	Immunoglobulin A
IR	insulin resistance
KO	knock-out
LD	lipid droplet
LDL	low-density lipoprotein
Lyso-PI	lyso-phosphatidylinositol
LOX-1	lectin-like oxidized LDL receptor-1
LPIAT1	lyso-phosphatidylinositol (Lyso-PI) acyl-transferase1
LPS	lipopolysaccharides
MAT	methionine adenosyltransferase
MBOAT7	membrane bound o-acyltransferase domain-containing 7
MC4R	melanocortin 4 receptor-deficient mice
miRNA	microRNA
mtDNA	mitochondrial DNA
MT-ND6	mitochondrially encoded NADH dehydrogenase 6
MTTP	microsomal triglyceride transfer protein large subunit gene
NAFLD	nonalcoholic fatty liver disease
NASH	nonalcoholic steatohepatitis
NF-κB	nuclear factor kappa-light-chain-enhancer of activated B-cells
NLRs	intracellular nucleotide-binding and oligomerization domain (NOD)-like receptors
OR	odd ratio
OS	overall survival
PAMPs	pathogen-associated molecular patterns
PD-1	death protein 1
PGC-1α	peroxisome proliferator-activated receptor γ coactivator-1α
PI	phosphatidylinositol
PNPLA3	patatin-like phospholipase domain-containing 3
PPARs	peroxisome proliferator activating receptors
PRSs	polygenic risk scores
PTEN	phosphatase and tensin homolog
PUFAs	polyunsaturated fatty acids
ROC	receiver operating characteristics
ROS	reactive oxygen species
RR	relative risk
SAM	S-adenyl methionine
SASP	senescence-associated secretory phenotype
SERCA	sarco/ER calcium ATPase
SIRTs	sirtuins
SNP	single nucleotide polymorphism
SR-A	scavenger receptor
SREBP1	Sterol Regulatory Element Binding Transcription Factor 1
STZ	streptozotocin
STAT3	Signal Transducer and Activator of Transcription 3
T2D	type 2 diabetes
TERT	telomerase reverse transcriptase
TG	triglyceride
TGF-β	transforming growth factor β
TLRs	toll like receptors
TM6SF2	transmembrane 6 superfamily member 2
TMC4	transmembrane channel like 4
TNF-α	tumor necrosis factor α
uPA	urokinase plasminogen activator
UPR	unfolded protein response
VLDL	very-low density lipoproteins
WD	Western diet
WNT	wingless
